# Molecular and Antigenic Characterization of *Piscine orthoreovirus* (PRV) from Rainbow Trout (*Oncorhynchus mykiss*)

**DOI:** 10.3390/v10040170

**Published:** 2018-04-02

**Authors:** Kannimuthu Dhamotharan, Niccolò Vendramin, Turhan Markussen, Øystein Wessel, Argelia Cuenca, Ingvild B. Nyman, Anne Berit Olsen, Torstein Tengs, Maria Krudtaa Dahle, Espen Rimstad

**Affiliations:** 1 Department of Food Safety and Infection Biology, Faculty of Veterinary Medicine, Norwegian University of Life Sciences, 0454 Oslo, Norway; dhamokan@nmbu.no (K.D.); oystein.wessel@nmbu.no (Ø.W.); turhan.markussen@nmbu.no (T.M.); ingvild.nyman@nmbu.no (I.B.N.); torstein.tengs@nmbu.no (T.T.); 2 National Institute of Aquatic Resources, Technical University of Denmark, 2800 Kgs. Lyngby, Denmark; niven@vet.dtu.dk (N.V.); arcun@vet.dtu.dk (A.C.); 3 Norwegian Veterinary Institute, 5003 Bergen, Norway; anne-berit.olsen@vetinst.no; 4 Norwegian Veterinary Institute, 0454 Oslo, Norway; maria.dahle@vetinst.no

**Keywords:** *Piscine orthoreovirus*, heart- and skeletal muscle inflammation, PRV-3, rainbow trout

## Abstract

*Piscine orthoreovirus* (PRV-1) causes heart and skeletal muscle inflammation (HSMI) in farmed Atlantic salmon (*Salmo salar*). Recently, a novel PRV (formerly PRV-Om, here called PRV-3), was found in rainbow trout (*Oncorhynchus mykiss*) with HSMI-like disease. PRV is considered to be an emerging pathogen in farmed salmonids. In this study, molecular and antigenic characterization of PRV-3 was performed. Erythrocytes are the main target cells for PRV, and blood samples that were collected from experimentally challenged fish were used as source of virus. Virus particles were purified by gradient ultracentrifugation and the complete coding sequences of PRV-3 were obtained by Illumina sequencing. When compared to PRV-1, the nucleotide identity of the coding regions was 80.1%, and the amino acid identities of the predicted PRV-3 proteins varied from 96.7% (λ1) to 79.1% (σ3). Phylogenetic analysis showed that PRV-3 belongs to a separate cluster. The region encoding σ3 were sequenced from PRV-3 isolates collected from rainbow trout in Europe. These sequences clustered together, but were distant from PRV-3 that was isolated from rainbow trout in Norway. Bioinformatic analyses of PRV-3 proteins revealed that predicted secondary structures and functional domains were conserved between PRV-3 and PRV-1. Rabbit antisera raised against purified virus or various recombinant virus proteins from PRV-1 all cross-reacted with PRV-3. Our findings indicate that despite different species preferences of the PRV subtypes, several genetic, antigenic, and structural properties are conserved between PRV-1 and-3.

## 1. Introduction

Rainbow trout (*Oncorhynchus mykiss*) is farmed in a variety of aquaculture systems in many countries [1]. Traditionally, rainbow trout is farmed in freshwater systems for production of portion size fish (300 g), while larger fish (3–5 kg) can be produced when fish are kept in seawater for the major grow-out period. In Norway, rainbow trout is primarily produced in seawater, and the loss of fish through the seawater stage has been estimated to 19% of the fish, mostly linked to infectious diseases [2].

In 2013, a new infectious disease was reported in rainbow trout in Norway. The disease occurred in freshwater hatcheries lasting until after sea transfer, and was characterized by lesions resembling those of heart and skeletal muscle inflammation (HSMI) in Atlantic salmon (*Salmo salar*), and by anemia [3]. HSMI in Atlantic salmon is caused by *Piscine orthoreovirus* (PRV) [4,5]. Screening for pathogens in the diseased rainbow trout revealed the presence of a PRV-like virus, and the nucleotide sequence that was obtained for a 562 nt region of the S1 genomic segment revealed 85% identity to PRV [3]. Experimental challenge studies in rainbow trout using blood as infective material, showed efficient viral replication in blood cells and in heart [6]. Recently, a PRV that was genetically similar to the rainbow trout PRV was reported in Coho salmon (*Oncorhynchus kisutch*) in Chile, suffering from an HSMI-like disease [7].

HSMI was first reported in Atlantic salmon in Norway in 2004, and occurs primarily in the marine phase. The disease leads to low to moderate mortality (0–20%), but has usually close to 100% morbidity [8]. The fish appear lethargic and anorectic and the histopathological changes include mild to severe inflammatory changes in the compact and spongy layer of the myocardium with similar, but milder changes in red skeletal muscle [9]. Disease outbreaks were initially reported to occur primarily five to nine months after seawater transfer [10], but now commonly occur earlier with an increasing number being detected already in the fresh water stage prior to sea water transfer [2].

PRV is recognized as a species of the genus *Orthoreovirus*, sub-family *Spinareovirinae*, family *Reoviridae*. It is a non-enveloped, icosahedral non-fusogenic virus with a double-stranded RNA (dsRNA) genome consisting of 10 linear segments L1-3, M1-3, S1-4 [4,11]. PRV is ubiquitous in seawater farmed Atlantic salmon, but has also frequently been detected in apparently healthy wild Atlantic salmon and in sea trout (*Salmo trutta*) [12]. The causality between a Norwegian PRV isolate and HSMI in Atlantic salmon was recently proven [5], but others report experimental PRV infection without pathology [13]. No viral genetic markers that were linked to virulence have been identified so far. In farmed Atlantic salmon, PRV is now found to be present from freshwater pre-smolts until slaughter, and the prevalence of infection increases after the sea transfer [14]. PRV infection in Atlantic salmon and cases of HSMI have also been reported from Scotland, Chile, and western North America [7,15,16,17].

A third variant of PRV, named PRV-2, was recently recognized as the causative agent of erythrocytic inclusion body syndrome (EIBS) in Coho salmon in Japan [18]. As the name of the disease implies, erythrocytes are the main target cells for PRV-2, which are similar to PRV in Atlantic salmon. The genomic organization of the Coho salmon PRV closely resembles that of the Atlantic salmon PRV. Comparisons at the whole genome level revealed however that the Coho salmon virus is genetically distinct, with a nucleotide identity of 73.4% in the coding regions [18]. In addition, a virus resembling PRV has been isolated from wild freshwater fish, largemouth bass (*Micropterus salmoides*), during a disease outbreak, and named large mouth bass reovirus (LMBRV) [19].

In this study, we have sequenced and analyzed the coding regions and the protein sequences of the PRV variant infecting rainbow trout, and analyzed the antigenic properties of this virus when compared to the PRV variant causing HSMI in Atlantic salmon. The phylogenetic analyses revealed that the rainbow trout PRV is genetically different from Atlantic salmon PRV. In line with the nomenclature used for the PRV infecting Coho salmon in Japan, i.e., PRV-2, we propose to name the PRV subtype infecting Atlantic salmon PRV-1, and the PRV subtype infecting rainbow trout PRV-3 (previously named PRV-Om).

## 2. Materials and Methods

### 2.1. Challenge Experiments

An *in vivo* experiment to generate PRV-3 positive material was carried out in the experimental facilities at DTU-VET in Denmark in accordance with the recommendations that are outlined in the current animal welfare regulations, under license No. 2013-15-2934-00976. The experimental protocols were approved by the Danish Animal Research Authority. The health status of the fish and environmental conditions were monitored on a daily basis during the experiments. Rainbow trout eyed eggs were provided by a commercial Danish fish farm that is officially free of infectious pancreatic necrosis virus (IPNV), infectious hematopoietic necrosis virus (IHNV), viral hemorrhagic septicemia virus (VHSV), and bacterial kidney disease (BKD). Following disinfection with iodine, the fish eggs were hatched and grown in the wet laboratory facilities at the European Union Reference Laboratory for fish disease (EURL, Copenhagen, Denmark) in UV-disinfected, recirculated tap water. Prior to infection, the rainbow trout were moved into a high containment facility harboring flow-through fresh water system, with a temperature of 12 °C ± 1 °C. For the production of challenge material, fish (*n* = 15) with an average weight of 270 g were anesthetized in water containing benzocaine (80 mg/L, Sigma) and injected i.p. with 0.1 mL homogenized blood cell pellet from PRV-3 infected fish diluted 1:4 (*v*/*v*) in L-15 medium. The virus isolate (NOR/060214) originated from a rainbow trout hatchery in Norway. The PRV-3 levels were monitored weekly by non-lethal blood sampling from five fish, which were marked by clipping of the adipose fin to avoid repeated sampling of the same fish. At three weeks post challenge (wpc), all of the fish were euthanized by immersing fish in water containing high concentration of benzocaine (800 mg/L). Blood was collected in heparin tubes, tested for PRV-3 levels by RT-qPCR, and stored at 4 °C. Plasma samples from the blood pellet were used for Illumina sequencing.

Another challenge trial was carried out in the NMBU aquarium research facility in Oslo, Norway in accordance with the recommendations of current animal welfare regulations, and the protocols were approved by the Norwegian Animal Research Authority. Rainbow trout from AquaGen AS (*n* = 22), average weight of 580 g were used. Upon challenge, the fish were anesthetized, as described above, and i.p. injected with 0.1 mL lysate from PRV-3-infected blood cells (1:3 dilution) originating from the previous experiment (Ct 22.8). The fish were reared in 500 L tanks with flow through freshwater and hand-fed a commercial diet (Skretting, Stavanger, Norway), at a rate of 2% of calculated biomass/tank/day. The PRV-3 viral load was determined weekly by RT-qPCR of blood collected by non-lethal blood sampling from the caudal vein of three anesthetized fish, which were marked by clipping of the adipose fin to avoid repeated sampling. When the Ct level for PRV-3 in 100 ng blood cell RNA were below lower than 25, the fish were euthanized, and blood collected on heparinized tubes. PRV-3 was purified from two blood samples (Ct 17.8 and 19.7). Purified virus was used for transmission electron microscopy and western blotting.

### 2.2. Virus Purification

Purification of PRV particles was performed, as previously described using CsCl density gradient and optimized for PRV from heparinized salmon blood sample [5,20]. In brief, 0.5 mL heparinized blood was mixed with 4.5 mL L15 medium and homogenized by sonication at 20 kHz for 30 s. Then, 10% sodium deoxycholate (SOC) was added (1:50), the samples were vortexed and left to stand for 5 min. This was repeated once, and samples were then incubated for 30 min on ice, emulsified with solvent vertrel XF, and centrifuged at 9000× *g* for 10 min at 4 °C to remove cell debris. The supernatant (4.2 mL) was layered over a CsCl gradient composed of 4.2 mL, 1.22 g/mL, and 4.2 mL 1.45 g/mL. Ultracentrifugation was performed at 30,000× rpm for 16 h, 4 °C using a SW 40TI rotor (Beckman Coulter, Brea, CA, USA) in an Optima LE 80K Ultracentrifuge (Beckman). Fractions of 0.5 mL were collected using a syringe with a 23 G needle. The density of the fractions was determined by cross referencing the refractive index [21]. The viral loads of all the fractions were estimated by RT-qPCR [5]. Fractions with a density corresponding to that of PRV-1 and low Ct values were chosen for dialysis. Samples were injected into Slide-A-Lyzer Cassette (G2 3.5 kDa MWCO, Thermo Fisher Scientific) and dialyzed at 4 °C with Dulbecco’s PBS without Mg or Ca (Sigma-Aldrich) for 1 h, 3 h, and then finally 12 h, separated by buffer changes.

### 2.3. Transmission Electron Microscopy (TEM)

Ten microliters of the dialyzed samples were used for TEM imaging. Samples were placed on paraffin film, and the 100 mesh carbon coated copper grids were placed over the drop for 1 min, washed 5× with distilled water, and stained with 4% aqueous uranyl acetate acid for 3 s. Excess liquid was removed and the grids were inspected in JEM 1400 Electron Microscope (JEOL Ltd., Tokyo, Japan), equipped with a TVIPS TemCam-F216 camera (TVIPS GmbH, Gauting, Germany).

### 2.4. RNA Isolation and RT-qPCR

Total RNA was isolated from pelleted blood cells, purified viral particles, and plasma. A total of 10 μL purified virus and plasma were diluted to 130 μL in PBS and added 420 μL Trizol LS (Life Technologies). For blood pellet, a volume of 20 μL was added to 650 μL Qiazol (Qiagen). The samples were homogenized in QIAzol Lysis Reagent using 5 mm steel beads and TissueLyser II (Qiagen) for 2 × 5 min at 25 Hz. After the addition of chloroform, the samples were centrifuged and the aqueous phase was transferred to RNeasy Mini spin column (Qiagen, Hilden, Germany). The RNA purification followed the manufacturer’s instructions. The RNA was stored at −80 °C. The RT-qPCR assays were performed using the Qiagen OneStep RT-PCR kit (Qiagen) adding 5 µl total RNA to each reaction tube, following the reaction conditions that were recommended by the manufacturer. The reverse transcription (RT) step was conducted at 50 °C for 30 min, followed by 95 °C for 15 min and 40 cycles of 94 °C/30 s, 55 °C/30 s, and 72 °C/30 s. The primers and probes used in the PRV-3 specific assay have previously been described [3].

### 2.5. Illumina Sequencing and Genome Assembly

Total RNA extracted from 2 mL of pooled plasma originating from two individuals (Ct 25.78 and 26), from challenge trial 1 was added 0.1 volumes of 3 M sodium acetate (pH 7.5) and 2X volumes of 100% ethanol, and mixed gently. Macrogen (Seoul, Korea) performed library preparation using the TruSeq RNA Library Prep Kit v2 (Illumina Inc., San Diego, CA, USA), followed by whole genome de novo sequencing (101 bases, paired-end reads) on an Illumina HiSeq4000 platform (1/7th lane). Sequences were de novo assembled using the genome assembler software SPAdes (version 3.10.1) [22].

### 2.6. Sequence and Phylogenetic Analyses

Multiple sequence alignments were performed using AlignX (Vector NTI AdvanceTM 11 package, InfoMax, Inc.) and phylogenetic analysis in MEGA7 software [23]. Pairwise nucleotide and amino acid sequence identities were calculated using the Sequences Identities and Similarities (SIAS) server (http://imed.med.ucm.es/Tools/sias.html). Protein secondary structure predictions were performed using PSIPRED v3.3, which is available at http://bioinf.cs.ucl.ac.uk/psipred/ [24]. The mVISTA methods of alignment was used for the comparison of concatenated complete coding sequences of PRV-3 genome segments with PRV-1, -2, and LMBRV [25]. Phylogenetic trees were constructed using the RNA-dependent RNA polymerase (RdRp) sequence obtained for PRV-3 in the present study, together with those from selected PRV-1 strains, PRV-2, Largemouth bass orthoreovirus (LMBRV), and representative orthoreoviruses from mammals, fish, birds, and reptiles. Maximum likelihood (ML) was used with the general time-reversible model of nucleotide substitution (best-fit substitution model that was suggested by the program) with gamma distribution and invariable sites [26]. Bootstrap values were calculated from 1000 replicates and values above 70 were considered to be significant [27,28]. 

Partial S1 sequences were obtained from PRV-3 strains from Europe (Denmark (3), Scotland (1), Germany (1) and Italy (2)) in 2017-2018 (Table S1). Primers PRV-3S1-ORF_F ATGGCGAACCATAGGACGGCGA and PRV-3-S1-ORF_R-TCACGCCGATGACCACTTGAGCA were used in PCR. Amplification was carried out using Qiagen OneStep RT-PCR Kit (Qiagen), according to the manufacturer’s instructions, with 25 pmol of each primer and 5 µl of template. RT-PCR conditions were 30 min at 50 °C, 15 min at 95 °C, 30 cycles consisting in 30 s at 94 °C, 30 s at 70 °C (−0.5 °C per cycle), and 1 min at 72 °C, followed by additional 30 cycles of 30 s at 94 °C, 30 s at 55 °C, and 1 min at 72, and a final extension at 70 °C for 10 min. PCR products were separated by electrophoresis on a 1.2% (*w*/*v*) agarose gel and the DNA bands obtained extracted and purified, following the protocol of the QIAquick Gel Extraction Kit (Qiagen). Sequencing was done by Eurofins Genomics (Germany) using the same primers as above.

Phylogenetic analysis of the partial S1 segment (876 nt) included the European PRV-3 strains, the Norwegian isolate (NOR/060214), and PRV sequences from Norway, Canada, Chile, and Japan were retrieved from GenBank (Table S1). Sequences were aligned by translation using MUSCLE v.3.8.425 [29]. ML tree was estimated with the RAxML v.8.2.11 package [30], using the GTM model with 1000 fast bootstrap replicates and 50% consensus Neighbor-Joining trees was also estimated, as implemented in Geneious v.11.0.2 (Biomatters Ltd., Auckland, New Zealand)

### 2.7. SDS-PAGE and Western Blot

Heparinized blood from rainbow trout challenged with PRV-3 collected at 0 wpc and 5 wpc were used in western blot (WB) analyses. Blood from Atlantic salmon, naïve or infected with PRV-1 were used as negative and positive controls, respectively [31]. Pelleted blood cells of equal volume were added Nonidet-P40 lysis buffer containing complete ultra mini protease inhibitor cocktail (1:5) (Sigma). Lysis was performed on ice for 30 min and the samples were then centrifuged at 5000× *g* for 5 min. Supernatant mixed with XT buffer and XT reducing agent (Bio-rad) were heated for 5 min at 95 °C and then loaded onto a 4–12% criterion XT bis-tris gel. Separated proteins were transferred onto a PVDF membrane and incubated overnight at 4 °C with antiserum against PRV-1 proteins; anti-σ1 (1:1000) [32], anti-σ3 (1:500) [13], anti-σNS (1:500) anti-µNS (1:1000) [33], anti-µ1C (1:500) [32], anti-λ1 (1:500), and anti-PRV-1 (1:25) (antiserum against purified PRV-1 particles) [5]. Horse radish peroxidase (HRP)-conjugated anti-rabbit IgG (Amersham, GE Healthcare, Buchinghamshire, UK) (1:20,000) was used as secondary antibody. The Clarity Western ECL Substrate kit was used for immunodetection (Bio-rad) and MagicMark as molecular weight ladder (XP Western Protein Standard, Invitrogen). Images were acquired using ChemiDoc XRS+ system and Image one software (Bio-rad).

## 3. Results

### 3.1. Morphology of Purified PRV-3 Viral Particles

**Figure 1:**
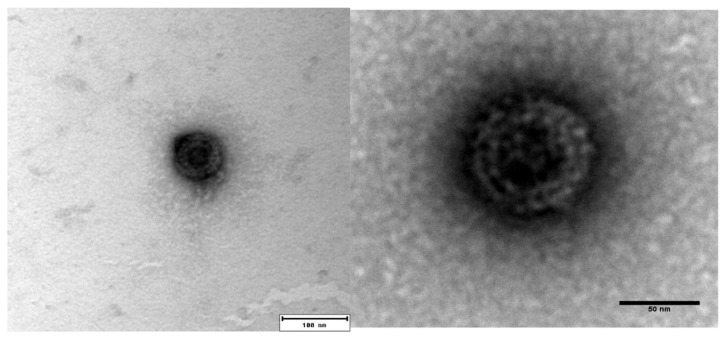
Transmission Electron microscope (TEM) image of negatively stained *Piscine orthoreovirus* (PRV)-3 showing approximately 75 nm diameter, spherical shaped virion.

PRV-3 infected blood pellet (Ct 17.8 and 19.7) that was harvested from rainbow trout at 5 wpc was used for purification. The CsCl gradient centrifugation did not yield a visible virus band, and fractions were therefore collected blindly. Fractions with densities between 1.35 and 1.33 g/cm^3^ were pooled and dialyzed. PRV-3 particles appeared in TEM as spherical, with an approximately 75 nm diameter. The icosahedral capsids had two concentric electron dense layers (Figure 1). The appearance of the viral particles are similar to from that previously observed for PRV-1 [5].

### 3.2. The PRV-3 Genome

The genome sequencing and analysis confirmed that PRV-3 is closely related to PRV-1. A total of 106,257,190 Illumina reads (53,128,595 pairs) were generated, and de novo assembly produced a large number of contigs. Several contigs had a high degree of amino acid sequence similarity to PRV-1. Careful examination of high coverage contigs revealed that the PRV-3 genome consists of 10 gene segments, similar to the orthoreoviruses.

Very high coverage was obtained for all segments, with average coverage ranging 430–652× (Table S2), but there was poorer coverage of the segment ends. These sequences could not be determined for all of the genomic segments. The complete 3’-end sequence was determined for segment L2 only, being identical to that of ortho- and aquareoviruses, (i.e., UCAUC-3’) [4,34,35]. The sequence at the 5’-ends were obtained for segments L1, L2, and S2, and was identical to that of PRV-1 (5’-GAUAAA/U), differing from other orthoreoviruses [4]. All the sequences of the coding regions of the PRV-3 genome were submitted to GenBank (MG253807-MG253816). 

**Figure 2:**
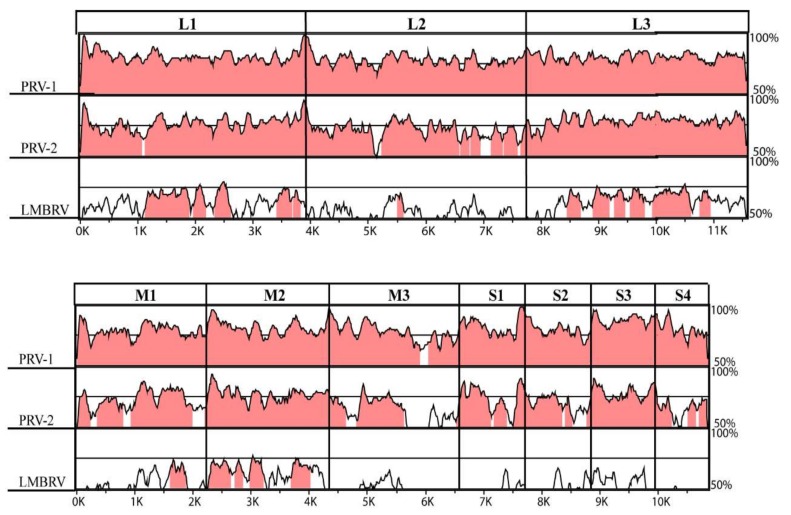
Stacked pair-wise conservation profile analysis. PRV-3 was used as a base sequence in an mVISTA alignment comparing whole genome segments with PRV-1, PRV-2, and LMBRV. Areas in pink colored regions illustrate >70% identities. The X-axis indicates the nucleotide sequence length in kb.

A detailed comparison of the PRV-3 coding sequences to PRV-1, PRV-2, and LMBRV was performed (Figure 2). The comparison showed conserved regions across L, M, and S segments among PRV, but only conservation between PRV and LMBRV was mainly seen for L1, L3, and M2. It has previously been reported that PRV-2 λ1, µ1, and p13 contain amino acid residue gaps when compared to PRV-1 [18]. The PRV-2 λ1 amino acid residue gap *(*T_13_*)* was shared by PRV-3. The remaining 10 open reading frames (ORFs) in the PRV-3 and PRV-1 genomes were of equal size.

Pairwise nucleotide- and amino acid sequence identities between PRV-1 and -3 were 80.1% (segment range 76.5–87.9%) and 90.5% (79.1–96.7%) for the nucleotide and the amino acid sequences, respectively. For PRV-3 versus PRV-2, the corresponding values were 72.9% (62.6–78.3%) and 80.0% (59.7–93.0%), respectively (Table 1). The amino acid identities of PRV-1 and PRV-3 were higher than the nucleotide identities for all the proteins, except σ3 and p13. 

### 3.3. High Conservation of Putative Functional Protein Domains between the Three PRV Subtypes

The RNA polymerase encoded by segment L1 displayed highest sequence conservation, while the µNS (M3), σ3 (S1), and σ1 (S4) proteins displayed the lowest (Table 1). Detailed analyses of all 11 known proteins that were encoded by PRV revealed that most previously predicted secondary structures and putative functional domains are conserved between PRV-3 and PRV-1 [11].

**Figure 3:**
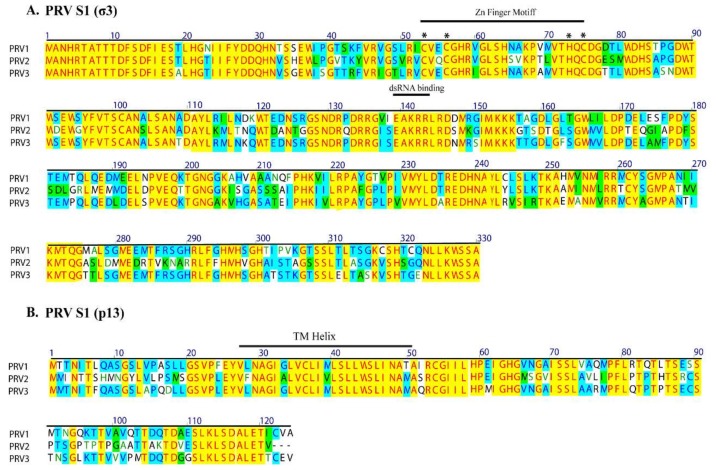
Alignment of deduced amino acid sequence of S1 segment of PRV-1, PRV-2, and PRV-3. The σ3 and p13 proteins are encoded in overlapping reading frames. The conserved transmembrane (TM) helix of p13 are encoded by the same region that encodes the conserved Zn finger motifs of σ3 (motifs labeled with *). Amino acid residues are numbered above. Identical sequence regions in all three PRVs are indicated by yellow background, identical amino acids shared by only two PRVs are in blue, and similar amino acids are shown with green background color.

The PRV S1 segment is bicistronic, encoding the outer capsid σ3 protein and a cytotoxic integral membrane protein, p13. The p13 is encoded by an overlapping internal reading frame of the S1 segment (nt 108–482). The predicted transmembrane helical region of p13, aa 26–48 is conserved among the PRVs. The conserved TM helix of p13 is encoded by the same region as the conserved Cx_2_Cx_16_Hx_1_C zinc-binding motif in the overlapping reading frame of the σ3 protein (Figure 3). Similarly, the conserved putative dsRNA binding helical region of σ3 also corresponds to a conserved part of p13. Notably, due to the restrictive nature of the overlapping reading frames, and conserved functional domains, the S1 segment nucleotide identity is higher than the amino acid identity/similarity between the PRVs. 

**Figure 4:**
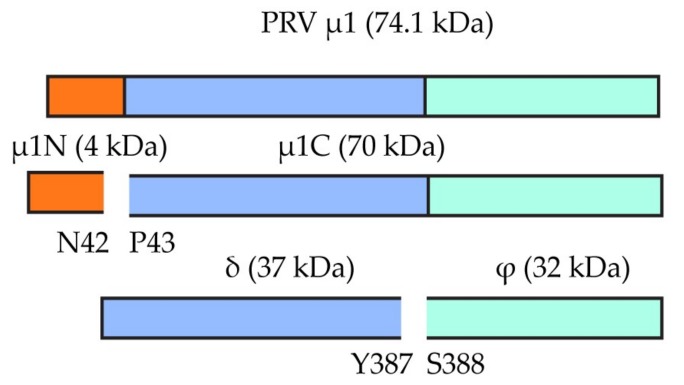
Putative cleavage sites of PRV µ1 protein. The top bar shows the full length µ1 protein, the middle bar shows the cleavage site of the N-myristoylated fragment µ1N and the bottom picture represent µ1C cleavage. The second cleavage site at position 387, which generates the δ/φ fragments, has a F387 for PRV-1, which in PRV-3 is replaced by Y387.

A sequence motif that differ between PRV-3 and PRV-1 was the putative cleavage site in the µ1 protein, where the µ1C is split in as µ1δ and µ1φ (Figure 4). This site was for PRV-1 suggested to be located at the position F387 [33], which in PRV-3 is replaced by Y387. The secondary structure prediction and structural comparison of the µ1 protein showed that PRV-3 µ1 protein lacks the aa 72–96 loop and the alpha helix (aa 279–295) as compared to *Mammalian orthoreovirus* (MRV) µ1 (Figure S1). The δ/φ cleavage site for MRV is located in the latter domain. 

### 3.4. Phylogenetic Analyses

**Figure 5:**
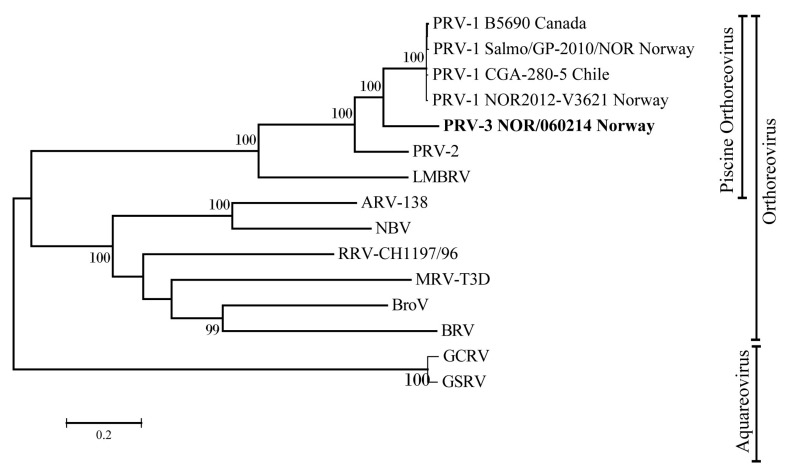
Phylogenetic tree based on complete coding sequences of PRV-3 RdRp (L1) of orthoreoviruses and aquareoviruses. MRV = *Mammalian orthoreovirus*, ARV = *Avian orthoreovirus*, NBV = *Nelson Bay orthoreovirus*, RRV = *Reptilian orthoreovirus*, BroV = *Broome virus*, BRV = *Baboon orthoreovirus,* GCRV = *Grass carp reovirus*, GSRV = *Golden shiner reovirus*.

The evolutionary relationship of PRV-3 to other PRV subtypes and members of the genera *Orthoreovirus* and *Aquareovirus* was determined by phylogenetic analysis. A phylogenetic tree was constructed using complete RNA dependent RNA polymerase (RdRp) coding sequence (Figure 5). The tree shows that piscine orthoreoviruses cluster separately from mammalian and avian orthoreoviruses. Within this cluster, a single monophyletic group was generated by PRV-1 strains with high bootstrap support. Corresponding phylogenetic trees were constructed for all the coding sequences, revealed similar tree topologies, placing PRV-3 closer to the PRV-1, than to the PRV-2 subtype (Figure S2).

**Figure 6:**
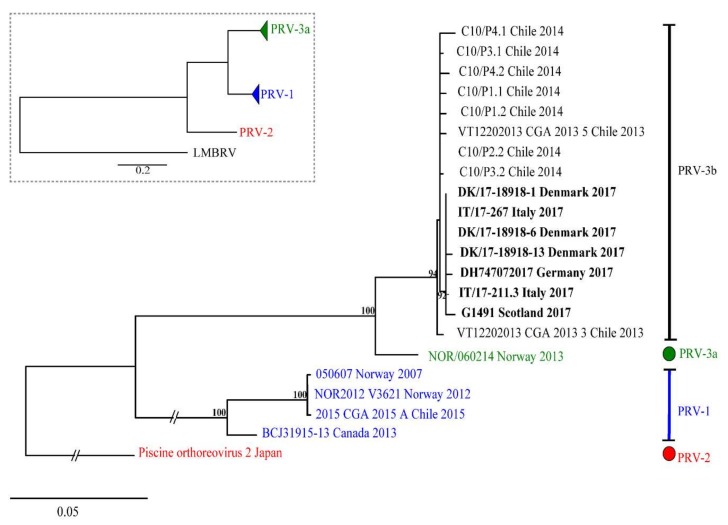
Phylogenetic analysis of partial nucleotide sequence of S1 segment of various PRV-3 isolates. The inner box represent the tree topography drawn in scale 0.5. Tree shows two PRV-3 clades, PRV-3a, and PRV-3b with strong support.

The results of pairwise comparison of nucleotide and amino acid sequences of S1 (σ3) are summarized in Table S3. The ML phylogenetic tree based on 876 nt of S1 segment showing the genetic relationship between available PRV-3 isolates is shown in Figure 6. In both ML and NJ phylogenetic analyses, PRV-3 is recovered as monophyletic group, with 100% bootstrap support. Within PRV-3, there are two well supported clades; one clade (3a) includes only the Norwegian isolate NOR/060214; whereas, the second clade (3b) includes sequences belonging to strains from different countries worldwide, including Chile, Denmark, Scotland, Germany, and Italy. Differences between PRV-3a and PRV-3b are 35 to 40 nt, or 6 to 10 aa in σ3 ORF. Within clade 3b, European sequences form a well-supported group (BS = 92%), different from the Chilean PRV-3 sequences. Within European sequences, differences of 0 to 2 aa are observed; whereas, the aa differences range from 2 to 7 aa when comparing the Chilean sequences with the European ones (Table S3). In summary, the three variants of PRV each form a monophyletic group.

### 3.5. Serological Cross-Reaction between PRV-1 and PRV-3

**Figure 7:**
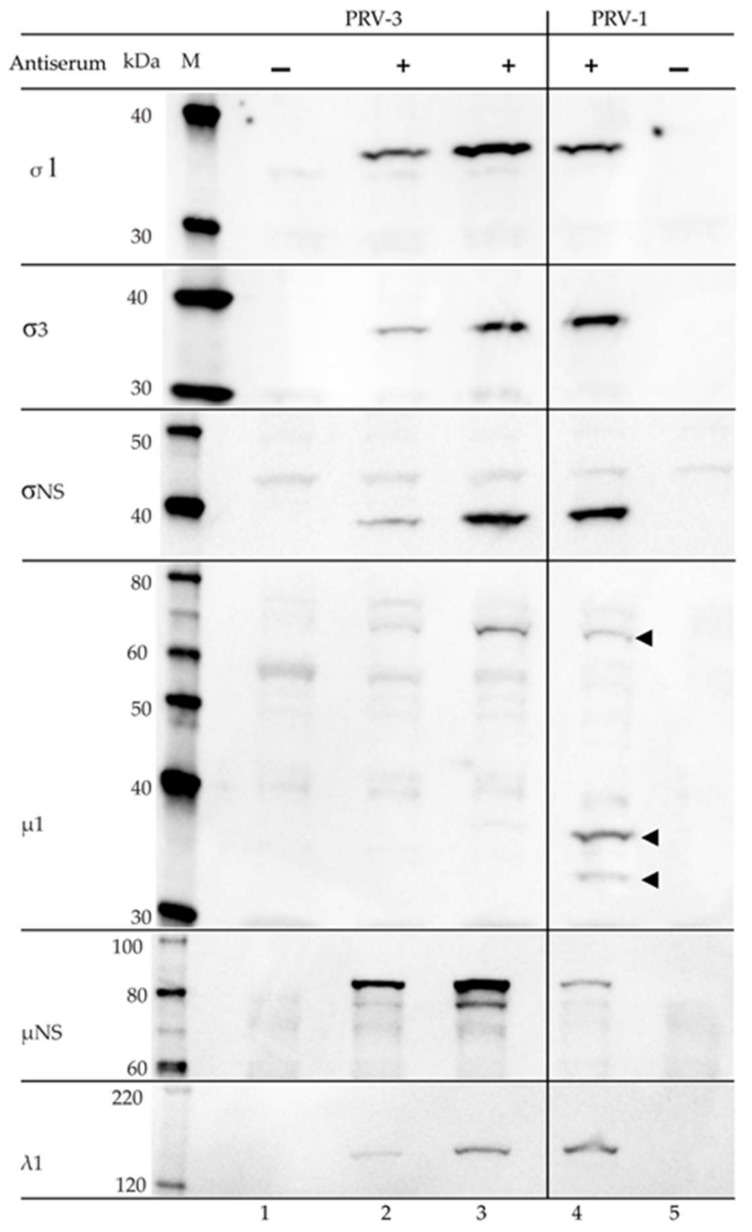
Western blots of PRV-3 and PRV-1 blood pellets using polyclonal antisera developed against recombinant proteins of PRV-1 σ1, σ3, σNS, μ1, μNS, and λ1. Lane 1-3 Rainbow trout: L1 negative control, L2-L3 PRV-3 infected samples. Lane 4-5 Atlantic salmon: L4 PRV-1 infected, L5: negative control. Arrowheads denote differing µ1 fragments in PRV-1.

The specificities of rabbit polyclonal antisera previously raised against the recombinant variants of PRV-1 σ3, σ1, σNS, µNS, µ1C, and λ1 were tested towards PRV-3 on western blot (Figure 7). The sera targeting the σ3 (outer capsid), σ1 (outer fiber), σNS (non-structural), and λ1 (core shell) all cross-reacted with PRV-3, producing bands that were corresponding to protein sizes of 35.6 kDa (σ3), 34.6 kDa (σ1), 39 kDa (σNS), and 141.5 kDa (λ1), respectively. The pattern of staining was identical to that observed for PRV-1. The antiserum targeting µNS identified two size variants from the PRV-3 sample, one of 83.5 kDa, and one weakly present at 76 kDa. Only the 76 kDa variant was seen for PRV-1 (Figure 7, lane 4), but two size variants have been observed in a previous study [33]. The 83.5 kDa band corresponds to the full-length protein. The 76 kDa band possibly represents secondary translation initiation at M_57_ (77.5 kDa) or M_85_ (74.4 kDa).

The antiserum against PRV-1 µ1 produced a 70 kDa band from PRV-3 infected blood cells on WB (Figure 7). Previous study of PRV-1 infected blood cells recognized four different variants of the µ1 protein, with sizes of 74.1, 70, 37, and 32 kDa, emerging at different stages during infection [33]. According to proteomic analysis of the PRV-1 µ1c fragments, the full-length PRV-1 µ1 protein of 74.2 kDa is most likely cleaved at N_42_P_43_ to form µ1N and the 70 kDa µ1C. The smaller fragments are likely to be generated following the second proteolytic cleavage of µ1C, possibly at F_387_S_388_, releasing the δ (37.7 kDa) and ϕ (32.1 kDa) fragments [33].

**Figure 8:**
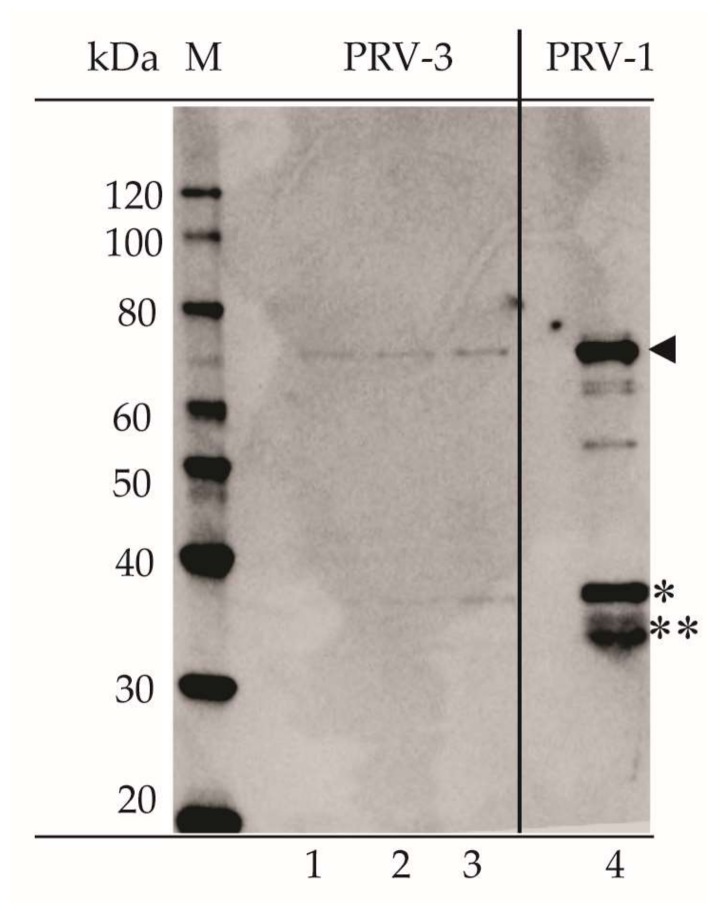
Purified PRV-3 (Lanes 1–3; Ct values for loaded virus particles were 22.7, 20.6 and 20.6, respectively) and PRV-1 (Lane 4; Ct value 17.1) particles analyzed by western blotting using antiserum against PRV-1. ◄ µ1, * σ3, ** σ1.

Rabbit antisera raised against purified PRV-1 particles produced bands of 70, 35 kDa in WB of PRV-1 virus particles, and similar for PRV-3 (Figure 8). The two bands most likely represent the outer capsid proteins of PRV, i.e., the µ1 (70 kDa), σ3 (37 kDa), and σ1 (35 kDa). In addition, more weakly stained bands were seen for purified PRV-1 corresponding to protein sizes of around 60 (apparent dual band) and 50 kDa.

## 4. Discussion

The aims of the present study were to perform genetic and antigenic characterization of the PRV variant recently identified in farmed rainbow trout. Complete coding nucleotide sequences of all the genomic segments were obtained from RNA isolated from plasma of infected fish. To classify as a new species within genus *Orthoreovirus*, the nucleotide sequence identities of homologous genome segments should be <60% equal to other orthoreoviruses, the amino acid sequence identities for more conserved proteins <65% and for more divergent outer capsid proteins <35% [36]. The new PRV from rainbow trout does not fulfill these criteria, thus it classifies as a *Piscine orthoreovirus,* together with PRV-1 and PRV-2. Furthermore, the 5′-terminal sequences are conserved for the particular orthoreovirus species. The 5′-terminal sequences were obtained for segments L1, L2, and S2 and were identical to those of PRV-1. We therefore propose the name PRV-3. 

Previous studies have shown that important structural and functional motifs in proteins of orthoreoviruses are highly conserved [11,37]. The PRV-1, PRV-2, and PRV-3 showed the highest sequence conservation in the L class segments, which encode proteins that are involved in transcription and replication, and in the core virus structure. The single amino acid (T_13_) gap observed in λ1 for both PRV-3 and PRV-2 when compared to PRV-1 is preceded by a highly basic N-terminal region (M_1_ERLKRKDKYKNT_13_…). It is interesting that the single aa gap is shared by two subtypes that were separated by large geographical distance and possibly differing host specificities. This may indicate a functional or structural role of this gap.

During the virus entry, the µ1 is autocleaved at N-terminal N_42_P_43_ and the release N-myristoylated fragment µ1N to facilitate core delivery [38,39]. This autolytic cleavage site residue is conserved between PRV and other orthoreoviruses [11]. In MRV, the second cleavage of the C-terminal µ1 generates the ϕ fragment, which is linked to optimal infectivity [40]. The third domain of MRV µ1 protein consists of five α helices and contain a protease cleavage site for δ-ϕ cleavage [41]. The differences in this second cleavage site and the generation of differently sized δ and ϕ fragments observed between PRV and MRV could be linked to structural differences that were observed in this region; the loop region aa 72–96 of MRV µ1 protein is shown to contribute to the stabilization of the capsids [42]. PRV, avian orthoreovirus (ARV), and aquareoviruses all lack this loop [43,44], which could be linked with evolutionary adaptation of the virus to different host species [42].

The lowest overall conservation for PRV-3 segments was observed for the M3 segment. The major gene product from this gene segment is the non-structural protein µNS, which is a multifunctional protein that is interacting with and recruiting other viral proteins to viral factories [45]. Although this segment has the lowest identity to other orthoreoviruses, it still has highly conserved predicted secondary structure [11]. The multifunctionality of µNS protein is likely a major contributing factor to the low conservation at the primary sequence level.

The phylogenetic analysis using the ORF for the RdRp showed that the fish orthoreoviruses, PRV1-3 and LMBRV form part of a larger monophyletic group that is separate from the other orthoreoviruses, with high bootstrap support. Within this larger fish orthoreovirus group, the PRVs form a monophyletic group. The phylogenetic analysis of the individual segments and of the concatenated sequences confirms that PRV-3 is genetically divergent and represents a new subtype of PRV.

The S1 sequence has high genetic homogeneity (96.1–100%) between PRV-1, but the identity between S1 of PRV-1 and PRV-3 was significantly lower (80.8%). Interestingly, the S1 segment of a PRV from Coho salmon in Chile with HSMI-like disease, together with sequences that were obtained from rainbow trout in Chile [7] cluster with the PRV-3 subtype and show high nucleotide identity to the clade of European PRV-3. Interestingly, PRV-3 has been detected in different countries in Europe both in clinical disease outbreaks in rainbow trout and in surveillance samples collected from healthy brown trout, which expands the list of susceptible species for this new PRV subtype.

A high conservation of protein structure in general between homologous PRV-1 and PRV-3 proteins were supported by secondary structure predictions. The ability of PRV-3 to infect both rainbow trout and Atlantic salmon and to target the same cell types as PRV-1 confirms the close relationship between PRV-1 and PRV-3 [6]. The cross-reaction of the PRV-1 σ1 antiserum with PRV-3 in western blot suggests that linear, but not necessarily conformational, epitopes of the outer fiber receptor-binding protein are conserved between PRV-1 and PRV-3. There are three serotypes in MRV and more than 11 serotypes that were described for ARV. For the serotyping of PRV, virus neutralization tests would have to be developed. This would require the ability to propagate the virus in cell cultures, which has not been successful for PRV so far.

Reoviruses are characterized by broad genetic diversity, which can be increased by their capacity of genome segment reassortment [46]. Investigation of the genetic diversity of PRV variants will aid in studies of pathogenesis of various host species and pathogen tracing. Atlantic salmon and rainbow trout are usually not stocked in the same farm, but can be present in different farms in close proximity. The occurrence of PRV-1 and PRV-3 infected fish in the dense and highly populated farming situations increase the risk of interactions between these PRV subtypes. The high level of sequence similarities, and the fact that PRV-3 can infect Atlantic salmon [6], increases the probability that new variants of PRV may evolve through the compatible reassortment of gene segments following a co-infection, as observed for ARV and MRV [47].

Reoviruses are ubiquitous in aquatic environment and are associated with fish diseases and mortality [19,48]. The finding of PRV variants in salmonid species other than Atlantic salmon have linked these PRV variants to diseases symptoms different from those of HSMI, in particular with anemia [3,18]. This indicates species-specific mechanisms of pathogenesis. Whether the species-specific disease characteristics are due to genetic differences in the PRV subtypes, salmonid host specific factors or a combination of both remains to be elucidated. The PRV-3 genome sequence that is reported in this study could be useful for studies of molecular pathogenesis and molecular epidemiology, and the observed antigenic relatedness between the PRVs could potentially be used for the development of vaccines and diagnostic methods. Future studies will aim to phenotypically characterize PRVs to better understand their tissue tropism, virulence, and possible re-assortment.

## 5. Conclusions

PRV-3 is a novel subtype of *Piscine orthoreovirus* reported from diseased rainbow trout. This is the first study describing the complete coding genome sequences of PRV-3 and the antigenic relationship between PRV-1 and PRV-3. The genome sequence was obtained by Illumina sequencing of plasma from infected fish. This study also reports for the first time that PRV-3 is present in several European countries.

## Figures and Tables

**Table 1:** Identities of nucleotide and amino acid sequences of ORFs of PRV-1, -2, and -3. ^a^ Protein nomenclature is based on PRV-1.

Segment	Protein Name ^a^	Predicted Function	PRV-3 & PRV-1		PRV-3 & PRV-2		PRV-1 &PRV-2	
			nt	aa	nt	aa	nt	aa
**L1**	**λ3 (Core RdRp)**	**RNA-dependent RNA polymerase**	**80.9**	**95.2**	**76.1**	**88.4**	**77.3**	**89.0**
**L2**	**λ2 (Core turret)**	**Guanylyltransferase, methyltransferase**	**77.8**	**90.0**	**70.9**	**77.1**	**71.1**	**76.9**
**L3**	**λ1 (Core shell)**	**Helicase, NTPase, RNA triphosphatase**	**80.3**	**96.7**	**78.3**	**93.0**	**77.5**	**92.7**
**M1**	**µ2 (Core NTPase)**	**NTPase, RNA triphos-phatase, RNA binding**	**78.4**	**88.7**	**72.0**	**78.3**	**72.2**	**78.1**
**M2**	**µ1 (Outer shell)**	**Outer capsid protein, membrane penetration**	**81.2**	**91.5**	**76.4**	**84.3**	**76.4**	**85.1**
**M3**	**µNS (NS-factory)**	**Non-structural protein**	**76.5**	**82.2**	**62.6**	**59.7**	**62.3**	**59.3**
**S1**	**σ3 (Outer clamp) **	**σ3: outer capsid protein, zinc metalloprotein**	**80.5**	**79.1**	**71.3**	**69.7**	**71.6**	**69.7**
	**p13 **	**p13: cytotoxic, integral membrane protein**	**85.6**	**78.2**	**78.1**	**63.7**	**77.3**	**62.9**
**S2**	**σ2 (Core clamp)**	**Inner capsid, RNA binding**	**80.4**	**88.8**	**70.1**	**73.8**	**70.2**	**77.1**
**S3**	**σNS (NS-RNA)**	**Non-structural protein**	**87.9**	**94.6**	**77.8**	**85.3**	**76.6**	**84.7**
**S4**	**σ1 (Outer fibre)**	**Cell attachment protein**	**80.0**	**81.6**	**64.5**	**64.4**	**65.5**	**66.7**
	**Concatenated coding sequences**		**80.1**	**90.5**	**72.9**	**80.0**	**73.4**	**80.3**

## Supplementary Materials

The following are available online at http://www.mdpi.com/1999-4915/10/4/170/s1: Figure S1: The secondary structure prediction and structural comparison of the µ1 protein; Figure S2: Phylogenetic trees constructed with genome segments of PRV-3; Table S1: List of sequences used for analysis and its NCBI accession numbers; Table S2: Number of reads targeting each PRV-3 segment from the Illumina HiSeq4000 run; Table S3: Nucleotide and amino acid variation between the partial S1 (nt 876) sequences of PRV isolates used in the phylogenetic analysis.
