# Supplementary material for: Molecular and Antigenic Characterization of Piscine orthoreovirus (PRV) from Rainbow Trout (Oncorhynchus mykiss)
Source: Viruses. 2018 Apr 2;10(4):170. doi: 10.3390/v10040170 (PMC5923464; doi:10.3390/v10040170)
Supplement: Supplementary file 1 [file viruses-10-00170-s001.pdf]

Supplementary Tables

Table S1. List of sequences used for analysis and its NCBI accession numbers.

Journal number.	Year	Species	Country	Subtype	GenBank Acc. No.
**NOR/060214**	2013	*Oncorhynchus mykiss*	Norway	PRV-3	MG983780
**DK/17-18918-1**	2017	*Oncorhynchus mykiss*	Denmark	PRV-3	MG983785
**DK/17-18918-6**	2017	*Oncorhynchus mykiss*	Denmark	PRV-3	MG983786
**DK/17-18918-13**	2017	*Oncorhynchus mykiss*	Denmark	PRV-3	MG983782
**G1491**	2017	*Oncorhynchus mykiss*	Scotland	PRV-3	MG983781
**773**	2017	*Oncorhynchus mykiss*	Germany	PRV-3	MG983787
**IT/17-211.3**	2017	*Salmo trutta fario*	Italy	PRV-3	MG983783
**IT/17-267**	2017	*Salmo trutta fario*	Italy	PRV-3	MG983784
**C10/P4.1**	2014	*Oncorhynchus mykiss*	Chile	PRV-3	KX844951
**C10/P1.2**	2014	*Oncorhynchus mykiss*	Chile	PRV-3	KX844964
**C10/P1.1**	2014	*Oncorhynchus mykiss*	Chile	PRV-3	KX844965
**C10/P4.2**	2014	*Oncorhynchus mykiss*	Chile	PRV-3	KX844959
**C10/P3.2**	2014	*Oncorhynchus mykiss*	Chile	PRV-3	KX844960
**C10/P3.1**	2014	*Oncorhynchus mykiss*	Chile	PRV-3	KX844961
**C10/P2.2**	2014	*Oncorhynchus mykiss*	Chile	PRV-3	KX844962
**VT12202013-CGA-2013-3**	2013	*Oncorhynchus kisutch*	Chile	PRV-3	KU131595
**VT12202013-CGA-2013-5**	2013	*Oncorhynchus kisutch*	Chile	PRV-3	KU131596
**BCJ31915_13**	2013	*Salmo salar*	British Columbia	PRV-1	KT429746
**050607**	2007	*Salmo salar*	Norway	PRV-1	KR337479
**2015-CGA-2015-A**	2015	*Oncorhynchus kisutch*	Chile	PRV-1	KU131604
**NOR2012-V3621**	2012	*Salmo salar*	Norway	PRV-1	KY429949

**PRV-2**	2012	*Oncorhynchus kisutch*	Japan	PRV-2	LC145616
**LMBRV**	2015	*Micropterus salmoides*	USA	PRV	KU974955
	2008	*Gallus gallus*	Canada	ARV	EU707935
	2011	*Pteropus poliocephalus*	Autralia	NBV	JF342673
**T3D**	2002	*Homo sapiens*	USA	MRV	HM159613
	2010	*Pteropus scapulatus*	Australia	BrOV	NC_014238
	1993	*Papio cynocephalus*	USA	BRV	NC_015878
**2511**	2015	*Eucampsipoda africana*	South Africa	MAHLV	NC_029912
	1979	*Notemigonus crysoleucas*	Canada	GSRV	AF403399
	2000	*Ctenopharyngodon idellus*	China	GCRV	AH009795
**CH1197/96**	2016	*Testudo graeca*	Switzerland	RRV	KT696549

Table S2. Number of reads targeting each PRV-3 segment from the Illumina HiSeq4000 run.

Segment	L1	L2	L3	M1	M2	M3	S1	S2	S3	S4
# of reads	**24936**	**20588**	**21162**	**10256**	**10733**	**10461**	**4378**	**5961**	**5213**	**4034**

Figure S1. The secondary structure prediction and structural comparison of the µ1 protein.

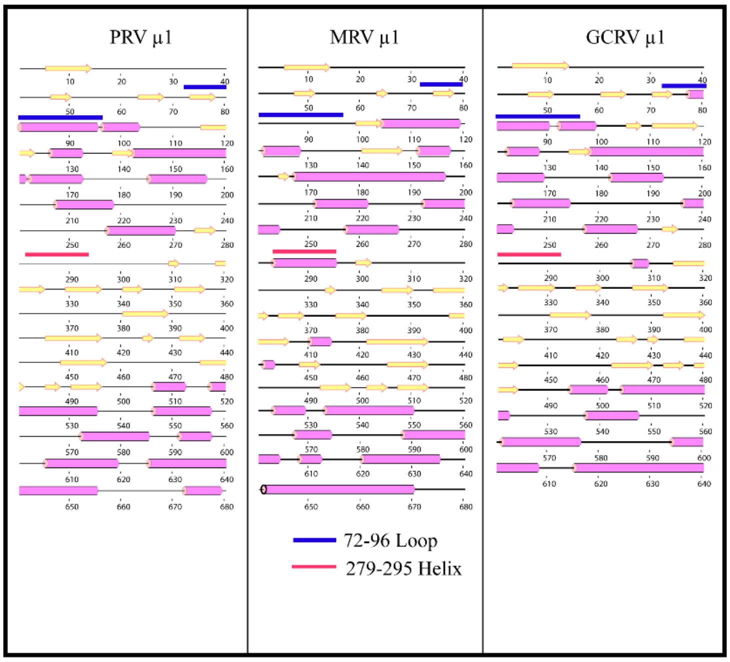

Figure S2. Phylogenetic trees constructed with genome segments of PRV-3.

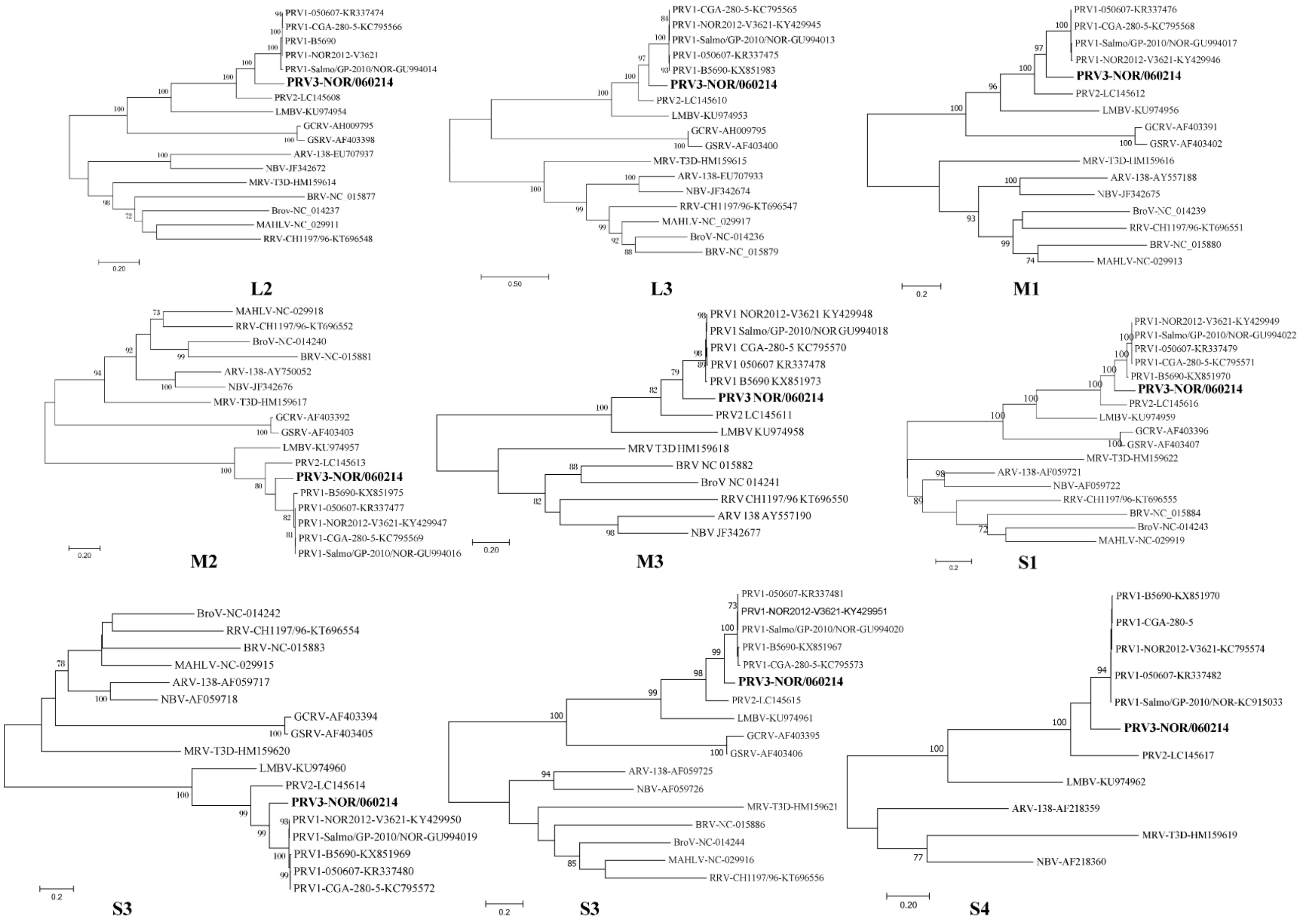

Table S3. Nucleotide and amino acid variation between the partial S1 (nt 876) sequences used in the phylogenetic analysis.

	LC145616 PRV-2 Japan	NOR/060214 Norway 2013	KX844951 C10/P4.1 Chile 2014	DH_747072017 Germany 2017	DK/17-18918-13 Denmark 2017	G1491 Scotland 2017	17-19266-27 Italy_2017	17-19266-35 Italy_2017	DK/17-18918-1 Denmark 2017	DK/17-18918-6 Denmark 2017	KX844964 C10/P1.2 Chile_2014	KX844965 C10/P1.1 Chile_2014	KU131595 VT12202013-CGA-2013-3 Chile_2013	KX844959 C10/P4.2 Chile_2014	KU131596 VT12202013-CGA-2013-5 Chile_2013	KX844960 C10/P3.2 Chile_2014	KX844961 C10/P3.1 Chile_2014	KX844962 C10/P2.2 Chile_2014	KT429746 BCJ31915_13 Canada_2013?	KR337479 050607 Norway_2007	KU131604 2015-CGA-2015-A Chile_2015	KY429949 NOR2012-V3621 Norway_2012
LC145616_Piscine orthoreovirus 2_ Japan		265	275	271	271	272	271	270	270	270	269	270	271	270	271	270	270	270	253	260	259	259
NOR/060214 Norway 2013	94		40	37	37	38	36	35	35	35	37	37	36	38	37	36	36	35	166	178	176	177
KX844951 C10/P4.1 Chile 2014	102	10		9	9	10	8	7	7	7	7	7	8	8	7	6	6	5	173	181	179	180
DH_747072017_ Germany_2017	98	7	7		4	5	3	2	2	2	6	6	7	7	6	5	5	4	172	180	178	179
DK/17-18918-13 Denmark 2017	97	7	7	2		5	3	2	2	2	6	6	7	7	6	5	5	4	171	178	176	177
G1491_Scotland _2017	97	6	6	1	1		4	3	3	3	7	7	8	8	7	6	6	5	171	179	177	178

17-19266-27 Italy_2017	97	6	6	1	1	0		1	1	1	5	5	6	6	5	4	4	3	171	179	177	178
17-19266-35 Italy_2017	97	6	6	1	1	0	0		0	0	4	4	5	5	4	3	3	2	170	178	176	177
DK/17-18918-1 Denmark 2017	97	6	6	1	1	0	0	0		0	4	4	5	5	4	3	3	2	170	178	176	177
DK/17-18918-6 Denmark 2017	97	6	6	1	1	0	0	0	0		4	4	5	5	4	3	3	2	170	178	176	177
KX844964 C10/P1.2_Chile _2014	97	8	6	5	5	4	4	4	4	4		4	5	5	4	3	3	2	170	178	176	177
KX844965 C10/P1.1_Chile _2014	98	8	6	5	5	4	4	4	4	4	4		5	5	4	3	3	2	169	177	175	176
KU131595 VT12202013-CGA-2013-3_Chile_2013	98	7	5	4	4	3	3	3	3	3	3	3		6	5	4	4	3	167	175	173	174
KX844959 C10/P4.2_Chile _2014	99	7	5	4	4	3	3	3	3	3	3	3	2		5	4	4	3	171	179	177	178
KU131596 VT12202013-CGA-2013-5_Chile_2013	99	7	5	4	4	3	3	3	3	3	3	3	2	2		3	3	2	169	177	175	176
KX844960 C10/P3.2_Chile _2014	99	7	5	4	4	3	3	3	3	3	3	3	2	2	2		2	1	168	176	174	175
KX844961 C10/P3.1_Chile _2014	98	6	4	3	3	2	2	2	2	2	2	2	1	1	1	1		1	167	175	175	174
KX844962 C10/P2.2_Chile _2014	98	6	4	3	3	2	2	2	2	2	2	2	1	1	1	1	0		168	176	174	175

KT429746 BCJ31915_13_Canada_2013?	92	63	65	63	63	62	62	62	62	62	63	62	61	62	62	61	61	61		36	36	35
KR337479 050607_Norway_2007	97	64	68	66	66	65	65	65	65	65	66	65	64	65	65	64	64	64	13		2	1
KU131604 2015-CGA-2015-A_Chile_2015	96	63	67	65	65	64	64	64	64	64	65	64	63	64	64	63	63	63	12	1		1
KY429949 NOR2012-V3621_Norway_2012	96	63	67	65	65	64	64	64	64	64	65	64	63	64	64	63	63	63	12	1	0	

Values above the diagonal are nucleotide differences and values below represents amino acid differences.
